# Personality Disorders in Persons with Gender Identity Disorder

**DOI:** 10.1155/2014/809058

**Published:** 2014-05-13

**Authors:** Dragana Duišin, Borjanka Batinić, Jasmina Barišić, Miroslav L. Djordjevic, Svetlana Vujović, Marta Bizic

**Affiliations:** ^1^Clinic of Psychiatry, Clinical Centre of Serbia, 11000 Belgrade, Serbia; ^2^Department of Psychology, Faculty of Philosophy, University of Belgrade, 11000 Belgrade, Serbia; ^3^Children's Hospital, School of Medicine, University of Belgrade, 11000 Belgrade, Serbia; ^4^Clinic of Endocrinology, Clinical Centre of Serbia, School of Medicine, University of Belgrade, 11000 Belgrade, Serbia

## Abstract

*Background.* Investigations in the field of gender identity disorder (GID) have been mostly related to psychiatric comorbidity and severe psychiatric disorders, but have focused less on personality and personality disorders (PDs). *Aims.* The aim of the study was to assess the presence of PDs in persons with GID as compared to cisgendered (a cisgender person is a person who is content to remain the gender they were assigned at birth) heterosexuals, as well as to biological sex. *Methods.* The study sample consisted of 30 persons with GID and 30 cisgendered heterosexuals from the general population. The assessment of PDs was conducted by application of the self-administered Structured Clinical Interview for DSM-IV Axis II PDs (SCID-II). *Results.* Persons with GID compared to cisgender heterosexuals have higher presence of PDs, particularly Paranoid PD, avoidant PDs, and comorbid PDs. In addition, MtF (transwomen are people assigned male at birth who identify as women) persons are characterized by a more severe psychopathological profile. *Conclusions.* Assessment of PDs in persons with GID is of great importance as it comprises a key part of personalized treatment plan tailoring, as well as a prognostic factor for sex-reassignment surgery (SRS) outcome.

## 1. Introduction

Persons diagnosed, according to Diagnostic and Statistical Manual of Mental Disorders 4th edition (DSM-IV) [1], with gender identity disorder (GID) suffer from strong, persistent discomfort between biological sex and experienced/expressed gender, with significant impairment in interpersonal, familial, social, professional, and other important areas of functioning. In DSM-V [2], in order to avoid stigma and to describe the stressed state of gender nonconformity, the diagnostic name is replaced with “gender dysphoria.” Sex-reassignment surgery (SRS) has proven to be an effective intervention for persons with GID, as confirmed by a number of follow-up studies reporting high levels of postsurgical satisfaction as well as improvement in the quality of life and the general functioning of patients who undertake it [3–6]. Presurgery diagnostic procedures in which psychiatric examination plays a major role are of great importance due to long-term consequences on the patient's life and functioning. The evaluation of SRS outcome is also important as SRS can also have negative implications, including personal regret and dissatisfaction, raising questions as to the most appropriate treatment (psychotherapy, cross-sex hormone treatment, and SRS) for persons diagnosed with GID [4, 7].

The review of the literature on the subject indicates that persons with GID have higher rates of psychological problems and psychiatric disorders, such as negative self-image, low self- esteem, adjustment disorders, depression, suicidality, and personality disorders (PDs) compared to normal controls [8–11]. Investigations in the field of GID have been mostly focused on the presence of psychiatric comorbidity and severe psychiatric disorders, as well as risks for suicidal behavior and self-mutilation [8, 9, 11, 12]. Studies focusing on the assessment of personality and PDs in gender dysphoric persons by standardized instruments are rare [9–17]. Review of the data in this domain has shown that assessment of psychopathology in persons with GID has been conducted at different phases of sex reassignment, predominantly with the following instruments: Minnesota Multiphasic Personality Inventory (MMPI-II), The Mini-International Neuropsychiatric Interview (M.I.N.I.), Structured Clinical Interview for DSM-IV (SCID), Defense Style Questionnaire (DSQ), Rorschach protocols, and so forth [12–16, 18–20].

The scientific literature regarding PDs in persons with GID offers contradictory results. Some studies have found the presence of PDs, while others did not find any PDs [8–16, 19, 21]. Discrepancies in results in some studies may be attributable to differing methodological issues (as shown in Table 4).

An overview of the literature offers data about relative prevalence rate of DSM-IV [1] Axis II disorders of between 3% and 66% [8–16], with cluster B PDs (borderline, histrionic, and narcissistic) identified as the most frequent, whereas some studies [13] have reported lower prevalence rates of PDs compared to higher rates recently found in large epidemiological samples [12, 16].

Certain researchers in the 1980s stressed the importance of psychological testing, addressing possible conceptualizations of GID (e.g., transsexualism) as a variant of borderline pathology [17]. GID, by some authors, has also been considered as a part of an underlying psychotic disorder [21], resulting in recommendations to abandon sex reassignment due to profound psychological dysfunction [22]. A third group of authors regard GID as a separate nosological entity, assuming psychiatric comorbidity as a consequence of persistent, sometimes long-life, gender incongruence and concomitant psychosocial distress [11, 16, 20]. Furthermore, psychiatric comorbidity and mental instability would appear to be important unfavorable prognostic factors for long-term psychosocial adjustment [3, 4, 23].

Although presence of PDs in persons with GID should not be considered an absolute contraindication for gender transition by cross-sex hormone therapy or SRS [16], it is reasonable to assume that the presence of any PDs may in fact interfere with adaptation to the postsurgical condition. Bodlund and Kullgren [4], for example, found that any PD diagnoses with a higher percentage of personality pathological traits were associated with negative postsurgery outcome. Despite clinical relevance, studies using standardized diagnostic instruments are rare.

An Italian study by Madeddu et al. [13] found the most frequent PDs from cluster B to be narcissistic PD, in particular, followed by histrionic and borderline PDs, with no differences between male-to-female (MtF) and female-to-male (FtM-transmen) (transmen are people assigned female at birth who identify as male) transsexuals (e.g., GID) samples.

Investigations by Bodlund et al. [14] found a statistically significant presence of PDs mainly within cluster B (assessed by SCID-II), as well as the majority with comorbid PDs in persons with GID. Furthermore, this study revealed significantly more subthreshold pathology among transsexuals (e.g., GID) than controls. The frequency of PD-fulfilled criteria was 29% among transsexuals (e.g., GID) versus 17% among controls [14].

The study of Haraldsen and Dahl [16], using SCID I and SCID-II for DSM-III-R and DSM-IV, found current Axis I disorders in 33% of 86 persons with transsexualism (e.g., GID) (predominantly mood and anxiety disorders) and Axis II disorders (e.g., PDs) (most frequently of cluster B) in 20% of persons. The authors have concluded that transsexual (e.g., GID) patients selected for SRS showed a relatively low level of self-rated psychopathology before and after treatment, suggesting that the view of transsexualism (e.g., GID) as a severe mental disorder is doubtful.

Cole et al. [11] reported that less than 10% of patients with GID evidenced problems associated with mental illness and that GID is usually an isolated diagnosis and not a part of any psychopathological disorder. Research by Hepp et al. [12] found high comorbidity in patients with GID, proposing psychiatric treatment in this respect. The authors found higher prevalence rates of Axis I disorders compared with the general population. The DSM-IV Axis II PDs diagnoses were found in all clusters as follows: clusters A (16,1%), B (22,6%), and C (19,4%) and PD not otherwise specified (6.5%).

A recent Japanese study by Hoshiai et al. [9] has registered psychiatric comorbidity in 19.1% male-to-female (e.g., transwomen) MtF and in 12.0% female-to-male (e.g., transman) FtM persons with GID and concluded that the majority of them had no psychiatric comorbidity. However, the aforementioned study showed that a high percentage (78%) of patients without current psychiatric comorbidity had seriously thought about committing suicide, with 30,6% of patients having performed self-mutilation [9].

The study of Miach et al. [19] did not find any parameters implying positive correlation between transsexualism (e.g., GID) and severe PDs.

We can summarize from the previously listed studies that GID is reported to be related to a high degree of personality psychopathology in the past, while recently, more studies imply that GID is an entity not related to severe psychiatric comorbidity.

The assessment of personality is one of the main predictive factors for the satisfied SRS outcome, according to previous studies [4, 5, 14, 24]. This is of crucial value not only in diagnosis of GID, but also even more in the assessment of psychological abilities and capacities in coping with the great difficulties of pretransitional and posttransitional life. It would be of great clinical and scientific value to present clinical cases of gender dysphoric persons in exploration phases [24] as well as through followup.

With this in mind, the aim of this study was to assess the presence of PDs in persons with GID compared to gender congruent heterosexual persons. The first hypothesis was that there was a significant difference in the presence of PDs between persons with GID and gender congruent heterosexual group. The second hypothesis was that there was also a significant difference in the presence of PDs in persons with GID with regard to biological sex.

## 2. Subjects and Methods

### 2.1. Sample and Procedure

The research was undertaken at the Clinic of Psychiatry, Clinical Centre of Serbia, over a two-year period. The study sample consisted of two groups: the first group consisted of 30 persons with GID (9 FtM and 21 MtF) with mean age 30.4 (min 19, max 49, SD ± 14.5). The second group consisted of 30 genderly congruent heterosexuals from the general population (15 females and 15 males) with mean age 35.07 (min 21, max 53, SD ± 14.9). Subjects with GID were explored in pretransitional, transitional, and posttransitional phase.

In both groups, inclusion criteria excluded current or past psychotic disorder. The diagnosis of GID was made by consensus of two board-certified psychiatrists according to the criteria of the 4th edition of the Diagnostic and Statistical Manual of Mental Disorders [1]. All subjects were interviewed individually by independent researchers at the clinical level taking medical and psychiatric histories, as well as family information, from which the researchers made their own diagnosis. The control sample of genderly congruent heterosexuals was randomly selected through voluntary and anonymous electronic interviews by placing selected testing materials on a website. At the time of the research, the medical authorities in Serbia did not require approval of the ethics committee. Participation of subjects was voluntary and anonymous.

### 2.2. Measures

The assessment of PDs was conducted by the self-administered Structured Clinical Interview for DSM-IV Axis II personality disorders (SCID-II) [25]. The SCID-II is a semistructured interview designed to provide categorical assessment (present or absent) of ten PDs, as well as depressive personality disorder, passive-aggressive personality disorder, and personality disorder not otherwise classified, which are included in Appendix B of DSM-IV [1]. The SCID-II was widely used both in research and clinical settings in the field of GID [12, 15, 16].

According to DSM-IV diagnostic criteria, SCID-II classified PDs in three clusters:

- cluster A: paranoid PD, schizoid PD, and schizotypal PD;
- cluster B: borderline PD, antisocial PD, narcissistic PD and histrionic PD;
- cluster C: avoidant PD, dependent PD, and obsessive-compulsive PD.

Modelled on the clinical interview, the instrument begins with a brief overview that characterized the subject's usual behaviour and relationships and provides information as to the subject's capacity for self-reflection. SCID-II has three columns: the left-hand column contains the interview questions, the centre column lists the DSM-IV diagnostic criteria, and the right-hand column is for recording item ratings. Each PD is rated as either “?,” “1,” “2,” or “3.” The “?” rating indicates that there is inadequate information to code the other three, so it should be recoded after the interview of family members or partners who are able to describe patterns of behavior. Applied to our sample, SCID-II showed good internal consistency (*α* = 0.87).

### 2.3. Data Analysis

In order to analyse obtained data we used the following statistical methods: *χ* ^2^ test, value Spearman correlation test, and discriminative analysis.

## 3. Results

### 3.1. Sociodemographic Characteristics of the Sample

The gender ratio was as follows: heterosexual group F : M = 15 : 15 (50 : 50%), GID group F : M = 9 : 21 (30 : 71%). There was not a significant difference in gender ratio (*P* = 0.114 > 0,05).

Mean age between the GID and cisgender heterosexual groups differs significantly (mean age in heterosexual group was 35.13 years and 29.77 years in group with GID (*P* = 0.007 < 0,05).

The participants in the two groups were all selected from an urban environment. The educational levels of the groups were as follows: elementary school/secondary school/high school: heterosexual group versus GID group: 0%/30%/70% versus 10%/63,30%/26,7%. The groups differ significantly regarding educational level (*P* = 0.000 < 0,05). The lower percentage of persons with GID who had completed high school could be explained by the fact that further education for persons with GID with new ID in Serbia is possible only after they pass surgical transition.

#### 3.1.1. Comparative Statistical Analysis of Presence of PDs in Persons with GID versus Cisgender Heterosexuals

As shown in Table 1, 20 out of 30 persons with GID compared to 11 out of 30 cisgender heterosexuals have PDs, which is of statistical significance (*P* = 0.029 < 0.05). Six persons (20%) with GID were diagnosed with one PD, as well as 6 (20%) heterosexuals. More than one PD (e.g., comorbid PDs) was found in 14 (46,66%) persons with GID and in 5 (16,7%) heterosexuals, which is of statistical significance (*P* < 0,05). The SCID-II results have shown a significant difference between the two groups in relation to presence of specific PDs as follows: 26,7% of persons with GID met the criteria for diagnosis of avoidant PD (*P* = 0,007) and 43,3% of paranoid PD (*P* = 0.000).

#### 3.1.2. Comparative Statistical Analysis of Presence of PDs in Persons with GID versus Cisgender Heterosexuals Related to Male Sex (Male—M)

As shown in Table 2, 23,8% of MtF from GID group (transwomen) met the criteria for diagnosis of avoidant PD compared to 0 (0%) in the male heterosexual group (*P* = 0,015).

Significant difference was found regarding presence of paranoid PD in the group with GID persons related to male biological sex as follows: MtF persons 9 (42,9%) (*P* = 0,011) compared to 1 (6,7%) in male heterosexuals.

Significant difference was found regarding the presence of schizoid PD—present in 4 MtF persons (19,0%) (*P* = 0.031) versus male heterosexuals with 0 (0%).

There was a significant difference in the presence of borderline PD related to male biological sex—present in MtF persons with GID in 38,1% (*P* = 0,001) compared to 0 (0%) in male heterosexuals.

We did not find any other significant difference in present PDs in relation to male sex when both groups were compared (as shown in detail in Table 2).

#### 3.1.3. Comparative Statistical Analysis of Presence of PDs in Persons with GID versus Cisgender Heterosexuals Related to Female Sex (Female—F)

Significant difference regarding presence of PDs in relation to female sex was found in paranoid PD, present in 4 FtM persons with GID (44,4%), while it was present only in one heterosexual female (6,7%) (*P* = 0.028). There was not any other significant difference in this respect, as shown in detail in Table 3.

## 4. Discussion

The most frequent DSM-IV [1] Axis II personality disorders assessed by SCID-II in our sample of persons with GID were from cluster A, paranoid PD, followed in order of relevance by cluster C, avoidant PD. Some of these data are similar and some are different from previous studies which have found cluster B as the most frequent in persons with GID, followed by clusters C and A [11–16]. Further analysis of obtained data has shown a significant presence of comorbid PDs in persons with GID, and the presence of comorbidity of PDs differentiates the two groups with statistical significance. Similar data were obtained in the study of Bodlund et al. [14], where 1/3 of patients who requested SRS had PDs from clusters B and C, while a majority of subjects had comorbid PDs.

A comparison with previous studies regarding psychiatric comorbidity on Axis II PDs among patients with GID (as shown in Table 4) indicates some differences in this study sample. High discrepancies in some studies (as shown in Table 4) related to the prevalence rate of Axis II PD diagnosis may be attributable to some methodological differences related to personality assessment methods. Some of the studies which used the SCID-II instrument have found different clusters of PDs such as PDs of all three clusters [12, 16] or PDs from clusters B and C [14, 15]. It is important to note that some studies which found high prevalence of clusters A and B or B and C diagnosis used clinical interview as a personality assessment tool [11, 14, 22]. The Italian study of Madeddu et al. [13] using DSM-IV criteria found cluster B PDs (narcissistic PD, followed by histrionic PD and borderline PD), cluster C diagnosis (obsessive-compulsive PD), NOS PD, and rarely PDs from cluster A.

Findings of significant presence of paranoid and avoidant PDs in the GID group of our study are not supported by findings in previous studies (as shown in Table 4). One of the possible explanations is sociocultural differences in Serbia, which could be tested in further investigations in the Balkans, in order to ascertain if there are certain discrepancies between northern and southern European countries in this respect.

The finding of paranoid and avoidant PDs as significant characteristics of persons with GID in our sample stresses the need for careful assessment of SRS candidates. These personality features strongly influence cognition and behavior of GID persons and therefore can be clinically relevant for eligibility and readiness for SRS.

Regarding the presence of PDs related to biological sex, our study found significant differences in Axis II PDs in persons with GID with regard to biological sex when compared with the same sex in the cisgender heterosexual group as follows: paranoid, schizoid, borderline, and avoidant PDs were significantly present in MtF persons compared to heterosexual males, while paranoid PD was significantly present in female sex within the group of persons with GID (FtM) compared to the female sex in the heterosexual group.

Our study is in line with most previous studies suggesting that MtF persons (transwomen) are generally characterized by a more severe psychopathological profile (23,26), when compared to FtM (transmen). Results of the Cole et al. study [11] also showed that psychiatric comorbidity is more frequent among MtF patients. On the other hand, there are some studies [13] not supporting the difference in PD prevalence with regard to biological sex. Haraldsen and Dahl [16] did not distinguish the frequencies of psychiatric comorbidity in MtF and FtM persons. Study of Madeddu et al. also [13] did not find any differences in psychopathological profile and severity between MtF and FtM persons.

Some authors have argued that there is a complex and still unclear relationship between the development of PDs and GID. Even though PDs and GID may be independent conditions, sometimes it may be difficult to establish whether GID symptoms could be better explained by personality disorder pathology. In some clinical cases it is mixed, and it is of great clinical relevance to indicate the presence of PD symptoms in GID persons [14] as the onset of both disorders can be traced back to adolescence/early adulthood. Clinical experience in persons with GID suggests that PDs may evolve as a dysfunctional way of coping with gender dysphoria [16, 26, 27].

A prevalence rate of Axis II disorders emerged, slightly higher than what was found in previous studies based on DSM-IV-oriented Structured Clinical Interviews [12, 15, 16]. The prevalence of DSM-IV Axis II disorders in general population based surveys, using diagnostic interviews, was between 4% and 13% [28], suggesting that individuals with GID are more prone to develop a PD. However, a personality disorder is not a precondition for developing a GID. The present study should enlighten the very underresearched issue of PD comorbidity in GID.

## 5. Conclusion

The study has confirmed two hypotheses—the first that there are significant differences in presence of PDs in persons with GID compared to genderly congruent heterosexual persons and the second that there are significant differences in the presence of specific PDs in persons with GID with regard to biological sex. A high percentage of comorbid PDs in persons with GID could be a consequence of overlapping of DMS-IV diagnostic criteria for PDs. According to DSM-V classification [2], it is recommended that a final diagnosis of GID should be confirmed in a six-month followup.

The authors of the study stressed the great importance of including personality assessment in standard GID diagnostic procedures, as presence or absence of PD** c**omorbidity is one of the contributing factors to the successful or unsuccessful SRS outcome [29].

## Figures and Tables

**Table 1:** Comparative statistical analysis of presence of PDs in GID persons versus cisgender heterosexuals.

PD type	Group		*P *
	GID *N* (%)	Heterosexual *N* (%)	
Avoidant	8 (26,7%)	1 (3,3%)	0.007*
Dependent	3 (10%)	1 (3,3%)	0.290 (n.s.)
Obsessive- compulsive	9 (30%)	5 (16,7%)	0.222 (n.s.)
Paranoid	13 (43,3%)	2 (6,7%)	0.001*
Schizoid	5 (16,7%)	1 (3,3%)	0.073 (n.s.)
Schizotypal	2 (6,7%)	0 (0%)	0.092 (n.s.)
Histrionic	5 (16,7%)	5 (16,7%)	0.999 (n.s.)
Narcissistic	4 (13,3%)	2 (6,7%)	0.385 (n.s.)
Borderline	10 (33,3%)	4 (13,3%)	0.067 (n.s.) (n.s.) > 0,05
Impulsive	3 (10,0%)	2 (6,7%)	0.639 (n.s.)
Antisocial	0 (0%)	0 (0%)	0.999 (n.s.)

*Significant.

n.s.: nonsignificant.

**Table 2:** Comparative statistical analysis of presence of PDs in GID persons versus cisgender heterosexuals related to male sex (Male—M).

PD type	Group/sex		*P *
	GID/MtF *N* (%)	Heterosexual/M *N* (%)	
Avoidant	5 (23,8%)	0 (0%)	0.015*
Dependent	2 (9,5%)	0 (0%)	0.135 (n.s.)
Obsessive- compulsive	6 (28,6%)	3 (20,0%)	0.555 (n.s.)
Paranoid	9 (42,9%)	1 (6,7%)	0.011*
Schizoid	4 (19,0%)	0 (0%)	0.031*
Schizotypal	2 (9,5%)	0 (0%)	0.135 (n.s.)
Histrionic	4 (4,8%)	2 (13,3%)	0.362 (n.s.)
Narcissistic	1 (4,8%)	1 (6,7%)	0.807 (n.s.)
Borderline	8 (38,1%)	0 (0%)	0.001*
Impulsive	3 (14,3%)	0 (0%)	0.064 (n.s.)
Antisocial	0 (0%)	0 (0%)	

*Significant.

n.s.: nonsignificant.

**Table 3:** Comparative statistical analysis of presence of PDs in GID persons versus cisgender heterosexuals related to female sex (Female—F).

PD type	Group/sex		*P *
	GID/FtM *N* (%)	Heterosexual/F *N* (%)	
Avoidant	3 (33,3%)	1 (6,7%)	0.093 (n.s.)
Dependent	1 (11,1%)	1 (6,7%)	0.707 (n.s.)
Obsessive- compulsive	3 (33,3%)	2 (13,3%)	0.250 (n.s.)
Paranoid	4 (44,4%)	1 (6,7%)	0.028*
Schizoid	1 (11,1%)	1 (6,7%)	0.707 (n.s.)
Schizotypal	0 (0%)	0 (0%)	0.092 (n.s.)
Histrionic	4 (44,4%)	3 (20,0%)	0.206 (n.s.)
Narcissistic	3 (33,3%)	1 (6,7%)	0.093 (n.s.)
Borderline	2 (22,2%)	4 (26,7%)	0.807 (n.s.)
Impulsive	0 (0%)	2 (13,3%)	0.159 (n.s.)
Antisocial	0 (0%)	0 (0%)	

*Significant.

n.s.: nonsignificant.

**Table 4:** An overview of the studies focusing on the presence of PDs and psychiatric comorbidity in persons with GID (e.g., transsexualism).

Author	Levine, 1980 [22]	Bodlund et al., 1993 [14]	Bodlund and Armelius, 1994 [15]	Cole et al., 1997 [11]	Haraldsen and Dahl, 2000 [16]	Hepp et al., 2005 [12]	Madeddu et al., 2009 [13]	Hoshiai et al., 2010 [9]	This paper
Diagnostic criteria	DSM- III	DSM-III-R	DSM-III-R	DSM-IV	DSM-III-R/IV	DSM-IV	DSM-III-R	DSM-IV	DSM-IV

Assessment method	Clinical interview	SCID	SCID screen	Clinical interview	SCID-II	SCID-II	SCID-II	—	SCID-II

Sex		MtF-10 FtM-9	—	MtF-318 FtM-117	MtF + FtM-86	MtF-20 FtM-11	MtF-34 FtM-16	MtF-230 FtM-349	MtF-21, FtM-9

Total sample	*N* = 51	*N* = 19	*N* = 18	*N* = 435	*N* = 86	*N* = 31	*N* = 50	*N* = 579	*N* = 30

Prevalence of PD	66%	3 (30%) 6 (67%)	33,3%	18 (6%) 5 (4%) 4% MtF, 3% FtM	8 (40%) 4 (36%) prevalence of PD 19,8%	42%	20 (77%) 23 (23%)	41 (19%) 38 (12%)	20 (66,6%) comorbid PDs 13 (46,6%)

Main results		Mainly cluster B or C PDs	Clusters B (22,2%), C (11,1%), and A (0%)	Clusters A (schizoid PD) and B (borderline PD) most frequent diagnosis	Clusters A 5,8%, B 8,1%, and C 5,8%, NOS 0%	Clusters A 16,1%, B 22,6%, and C 19,4%, NOS 6,5%	Clusters A 2%, B 22%, and C 12%. NOS 16% No differences in psychopathological profile and severity between MtF and FtM TS	Adjustment disorder (6,7%), anxiety disorder (3,6%), and mood disorder (1,4%, 8/579): all were associated with histories of suicidal ideation (in FtM, but not in MtF group)	Clusters A and C (paranoid PD—43,3% and avoidant PD—26,7%). Differences in relation to biological sex in GID group versus heterosexuals (MtF: paranoid, schizoid, borderline, and avoidant PD; FtM: paranoid PD)

Sample characteristics	Patients requesting SRS	Transsexuals, for example, GID (DSM-III-R)	Transsexuals, for example, GID (DSM-III-R)	Patients requesting SRS	Mixed sample, pre- and post-SRS	GID (DSM-IV)	GID (DSM-III-R)	GID (DSM-IV)	GID patients (DSM-IV)
